# Immunogenicity and safety of a respiratory syncytial virus fusion protein (RSV F) nanoparticle vaccine in older adults

**DOI:** 10.1186/s12979-017-0090-7

**Published:** 2017-04-12

**Authors:** Louis Fries, Vivek Shinde, Jeffrey J. Stoddard, D. Nigel Thomas, Eloi Kpamegan, Hanxin Lu, Gale Smith, Somia P. Hickman, Pedro Piedra, Gregory M. Glenn

**Affiliations:** 1 Novavax, Inc, 20 Firstfield Road, Gaithersburg, MD 20878 USA; 2 Baylor College of Medicine, One Baylor Plaza, Houston, TX 77030 USA

**Keywords:** Anti-F IgG, F or fusion protein, Microneutralization, Nanoparticle vaccine, Palivizumab-competitive antibody (PCA), Recombinant, Respiratory syncytial virus (RSV), Avidity

## Abstract

**Background:**

A preventative strategy for Respiratory Syncytial Virus (RSV) infection constitutes an under-recognized unmet medical need among older adults. Four formulations of a novel recombinant RSV F nanoparticle vaccine (60 or 90 μg RSV F protein, with or without aluminum phosphate adjuvant) administered concurrently with a licensed inactivated trivalent influenza vaccine (TIV) in older adult subjects were evaluated for safety and immunogenicity in this randomized, observer-blinded study.

**Results:**

A total of 220 healthy males and females ≥ 60 years of age, without symptomatic cardiopulmonary disease, were vaccinated concurrently with TIV and RSV F vaccine or placebo. All vaccine formulations produced an acceptable safety profile, with no vaccine-related serious adverse events or evidence of systemic toxicity. Vaccine-induced immune responses were rapid, rising as early as 7 days post-vaccination; and were comparable in all formulations in terms of magnitude, with maximal levels attained within 28 (unadjuvanted) or 56 (adjuvanted) days post-vaccination. Peak anti-F protein IgG antibody levels rose 3.6- to 5.6-fold, with an adjuvant effect observed at the 60 μg dose, and a dose-effect observed between the unadjuvanted 60 and 90 μg regimens. The anti-F response persisted through 12 months post-vaccination. Palivizumab-competitive antibodies were below quantifiable levels (<33 μg/mL) at day 0. The rise of antibodies with specificity for Site II peptide, and the palivizumab-competitive binding activity, denoting antibodies binding at, or in proximity to, antigenic Site II on the F protein, closely paralleled the anti-F response. However, a larger proportion of antibodies in adjuvanted vaccine recipients bound to the Site II peptide at high avidity. Day 0 neutralizing antibodies were high in all subjects and rose 1.3- to 1.7-fold in response to vaccination. Importantly, the RSV F vaccine co-administered with TIV did not impact the serum hemagglutination inhibition antibody responses to a standard-dose TIV, and TIV did not impact the immune response to the RSV F vaccine.

**Conclusions:**

RSV F protein nanoparticle vaccine induced increases in measures of functional immunity to RSV in older adults and demonstrated an acceptable safety profile. Adjuvanted formulations provided additional immunogenicity benefit as compared to increasing antigen dose alone. This trial was registered with ClinicalTrials.gov number NCT01709019.

## Background

Respiratory syncytial virus (RSV), the leading viral cause of severe lower respiratory tract disease in infants and young children worldwide, is increasingly being recognized as a significant cause of morbidity and mortality in older adults [1]. Although infants and children are usually infected by 2 years of age, the resultant immunity to RSV is relatively ineffectual and frequent reinfections occur throughout life. RSV infection in older adults generally begins in the upper respiratory tract, but progressively spreads to the lower respiratory tract in 90% of cases [2]. Adults with underlying risk factors may present with RSV-associated disease of increased severity and duration. RSV may also trigger clinical deterioration in frail older adults, the immunocompromised, and those with chronic cardio-pulmonary disease, resulting in RSV-associated hospitalization rates approaching those associated with influenza [2–4]. RSV is a predictable seasonal cause of respiratory illness that burdens the healthcare system, resulting in increased numbers of medical visits, hospitalizations, and deaths. Published estimates indicate that approximately 11,000 to 17,000 older adults die annually of RSV-related illnesses in the United States (US), with about 10-fold more (177,500) admitted to the hospital with respiratory symptoms [5, 6]. Vaccination against RSV has the potential to be a highly beneficial and effective approach to limit symptomatic RSV infection in older adults as well as other high-risk adult and pediatric populations.

A novel RSV F nanoparticle vaccine (herein termed RSV F vaccine) was developed based on a purified, recombinant, near-full-length RSV fusion (F) glycoprotein that has been demonstrated to be stable, well-tolerated, immunogenic, and fully protective in animal challenge studies [7, 8]. Clinical evaluation in healthy young adults has shown that both unadjuvanted and aluminum phosphate adjuvanted RSV F vaccine formulations were well-tolerated and induced robust antibody responses [9, 10], including the induction of antibodies with specificity to epitopes on the Site II domain- the target of broadly neutralizing monoclonal antibodies such as palivizumab and motavizumab.

The present study sought to evaluate the safety and immunogenicity of a single immunization with a 60 or 90 μg dose of RSV F vaccine, formulated with or without aluminum phosphate, in older adults, age 60 years and older. The dose-ranging component of this study was directed toward selection of a formulation and dose level to examine in future clinical trials that will also explore efficacy of the RSV F vaccine. Given that the annual RSV and influenza epidemics share similar seasonality, this study also sought to address the impact and potential for immunological interference between the RSV F vaccine and TIV when administered concurrently.

## Methods

### Study conduct

This study was conducted at four clinical sites in the US (Arizona, Florida, Texas, and Utah) from 12 October 2012 to 05 November 2013, in accordance with International Conference on Harmonisation Good Clinical Practice and the Declaration of Helsinki. The protocol and informed consent were reviewed and approved by a central institutional review board (Copernicus Group IRB, Research Triangle Park, NC), and all subjects provided written informed consent prior to any trial-related procedures. The trial was registered with ClinicalTrials.gov (NCT01709019).

### Study design

**Fig. 1:**
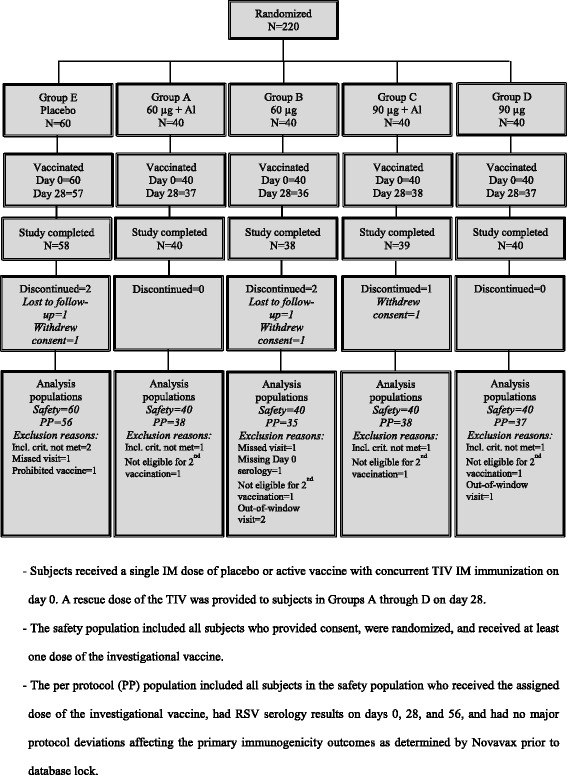
Subject disposition

This Phase 1, randomized, observer-blinded, placebo-controlled trial enrolled healthy male and female subjects ≥ 60 years of age, without symptomatic cardiopulmonary disease or risk factors known to increase the risk of influenza-related complications, including diabetes mellitus, congenital or acquired blood dyscrasias, renal or hepatic dysfunction, and morbid obesity; and who had not received any influenza vaccine within 3 months prior to study start. A total of 220 eligible subjects were enrolled and randomized in one of five treatment groups, with stratification by age (60 to < 75 years and ≥ 75 years) to distribute the proportion of persons in each age stratum equally across treatment groups. Treatments comprised a single intramuscular (IM) dose of a placebo or RSV F vaccine on day 0 into the left deltoid as described in Fig. 1, with concurrent TIV IM immunization in the opposite deltoid. Rescue TIV immunization was provided to active vaccine recipients in Groups A through D on day 28 (to control the risk that the initial dose with RSV F vaccine may have blunted the influenza hemagglutination inhibition [HAI] response), while subjects in Group E received a second dose of placebo. Study follow-up spanned approximately one year from dosing on day 0 for all subjects.

### Study vaccine

The RSV F vaccine was manufactured as previously described [7, 8]. The vaccine was formulated at two RSV F protein dose levels in a 25 mM phosphate buffer, pH 6.2, with 0.15 M NaCl, 1% histidine, and 0.01% Polysorbate (PS)-80 for unadjuvanted formulations; or in a 12.5 mM phosphate buffer, pH 6.2, with 0.15 M NaCl, 0.5% histidine, 0.005% PS-80, and 2.4 mg/mL of aluminum phosphate (AdjuPhos, Brenntag Biosector, Frederikssund, Denmark) for adjuvanted formulations. The licensed TIV, Fluzone® (Sanofi Pasteur, Swiftwater, PA), included the three influenza strains recommended for the 2012–2013 influenza season in the Northern hemisphere and was administered according to manufacturer instructions. The placebo consisted of isotonic saline.

### Immunogenicity measurements

Subjects provided blood samples on days 0, 7, 28, 56, 119, 182, and 364. Antibody concentrations/titers for the palivizumab-competitive antibody (PCA) and linear antigenic Site II peptide ELISAs; and the RSV/A and RSV/B microneutralization (MN) assays were determined as previously described [8–10]. For the anti-F IgG ELISA, serial dilutions of subject sera and an anti-F IgG reference standard were added to RSV F antigen-coated microtiter plates and incubated, followed by washing, and then the addition of anti-human IgG peroxidase conjugated antibody and the peroxidase substrate. A four-parameter logistic curve fit was applied to the reference standard and the reported result for each sample was determined from the mean calculated from serial dilutions, interpolated from the reference standard concentrations in arbitrary ELISA Units (EU). Day 0, 28, 56, and 182 sera were used for HAI titer determination for all three antigens contained in the TIV. Influenza hemagglutinin inhibition (HAI) testing was conducted at an initial dilution of 1:10 and followed by a series of 2-fold dilutions until a final dilution of 1:1,280 was reached. The appropriate virus antigen and indicator erythrocyte suspension was added to designated wells in two steps, with mixing and incubation at each step. The titration end-point was taken as the highest dilution that demonstrated complete inhibition (100%) of hemagglutination. The serum HAI titer was the geometric mean (GM) of duplicate test results.

### Surface plasmon resonance

Binding kinetic assays were performed according to standard kinetics application method using the Biacore™ T200 (GE Healthcare Life Science, Pittsburg, PA). In brief, biotinylated antigenic peptide (NSELLSLINDMPITNDQKKLMSNNV, synthesized by Peptide 2.0, Chantilly, VA) was immobilized onto a Sensor Chip SA (GE Healthcare Life Science) at 15–20 resonance units (RU) on the active flow cell. Human sera were subjected to centrifugal filtration, then diluted 1:50 into HBS-EP buffer (0.01 M HEPES pH 7.4, 0.15 M NaCl, 3 mM EDTA, 0.005% (v/v) Surfactant P20). An RSV negative serum sample (diluted 1:50, Valley Biomedical, Lot # E50790S) was used as the sample buffer control. Palivizumab served as the positive control. Samples were injected through the flow cell at 40 μl/min for 180 s; dissociation was recorded for 420 s. The chip surface was regenerated by injecting 100 mM HCl at 40 μl/min for 30 s between runs. Data were analyzed by kinetic analysis using 1:1 fitting; only k_off_ rates were analyzed. A k_off_ rate of 1 was assigned to any sample whose sensorgrams showed no detectable binding or had a dissociation rate too rapid to be determined.

### Safety measurements

Vaccinees were monitored for approximately 30 min post-injection for observation of immediate local or systemic reactions. Diaries were provided to all subjects to document any AEs (both solicited and unsolicited) and to record body temperature, concomitant medications, and medical visits and procedures, starting on each vaccination day and continuing for 6 days thereafter. A series of local injection site (pain, bruising, redness, and swelling) and systemic (oral temperature, chills, myalgia, arthralgia, diarrhea, nausea, vomiting, headache, and fatigue) reactions, reasonably likely to occur in vaccine programs, were solicited in the diary. AE severity grading was based on the FDA Draft Guidance for Industry, Toxicity Grading Scale (TGS) for Healthy Adult and Adolescent Volunteers Enrolled in Preventive Vaccine Clinical Trials (September 2007).

### Statistical analysis

The safety population was used for all safety analyses and was defined as all study subjects who provided consent, were randomized, and received any study test article. The Per Protocol (PP) population was used for all immunogenicity analyses and was defined as all subjects in the safety population who received the assigned dose of the investigational vaccine according to protocol and as randomized, had RSV serology results at least at days 0 (baseline), 28, and 56, and had no major protocol deviations affecting the primary immunogenicity outcomes as determined by Novavax prior to unblinding. The primary endpoint for the immunogenicity assessment was anti-F IgG EU, while the HAI titers against each of the influenza A and B antigens included in the TIV were the secondary endpoints. Other RSV serologic measures were exploratory endpoints.

Safety assessments were performed descriptively and included the number and percentage of subjects with solicited local and systemic AEs over 7 days post-dosing; all unsolicited AEs over 56 days post-dosing; and medically-attended events (MAEs), serious adverse events (SAEs), and significant new medical conditions (SNMCs) over one year post-dosing. Summaries of solicited local and solicited systemic AEs were presented by severity (mild, moderate, severe) and by vaccination window (days 0–6, days 28–34, or combined). Unsolicited AEs were summarized by Medical Dictionary for Regulatory Activities (MedDRA) system organ class (SOC) and preferred term (PT), and presented by severity and relationship. Clinical laboratory abnormalities reported as AEs were included in the summary of all AEs.

The primary objective of demonstration of an adjuvant effect was tested by examining the differences in the means of the log_10_-transformed day 28 and (separately) day 56 anti-F IgG EUs in subjects who received 60 or 90 μg RSV F antigen dose with or without adjuvant (Geometric Mean Ratio_Adj/Unadj_; GMR_Adj/Unadj_). In order to increase the power of this contrast between adjuvanted and unadjuvanted formulations, an analysis of pooled adjuvanted vaccine (Groups A + C) versus pooled unadjuvanted vaccine (Groups B + D) groups was performed, after testing for homogeneity of variance among groups, using analysis of covariance (ANCOVA) with baseline EU as a covariate. The same pooled analysis strategy was used to determine whether the 90 μg antigen dose could demonstrate an immunogenicity advantage over the 60 μg antigen dose, with or without aluminum phosphate (GMR90μg/60 μg). The effect of RSV F/TIV co-administration on resultant HAI titers (for each antigen included in the vaccine) was assessed descriptively, based on HAI Geometric Mean Titers (GMTs), Geometric Mean Ratios (GMRs), Seroconversion Rates (SCRs), and Seroprotection Rates (SPRs), with their 95% CIs. Seroconversion was defined as either a baseline HAI titer < 10 and a post-vaccination titer ≥ 40, or a baseline HAI titer ≥ 10 and ≥ 4-fold increase in post-vaccination HAI titer relative to baseline. Seroprotection level was defined as a post-vaccination HAI titer ≥ 40. All statistical analyses were two-tailed and assessed at the 5% significance level.

The GMTs or geometric mean concentrations (GMCs) and their associated 95% CIs were measured to describe the response profile for the exploratory immunogenicity endpoints (e.g., antigenic site II and PCA ELISAs, and RSV/A and RSV/B MN).

### Sample size

Given a total of 160 subjects receiving an RSV F vaccine in association with TIV, the study had an 80% probability of detecting at least one AE that occurs at a true rate of 1.0%, and an upper 95% confidence bound for incidence rate of 1.9% for any event which was not observed. This was selected as adequate and reasonable for an initial review of the safety profile, rather than for statistical reasons. For immunogenicity analyses, a sample size of 80 subjects in pooled RSV F vaccine groups would provide 88% power to detect a 2-fold difference in GMTs for detecting adjuvant effects. Power estimates were based on a log_10_ SD of 0.6 for anti-F IgG EUs, with an alpha of 0.05 using a two-sided two-sample *t*-test for significance determination.

## Results

### Subject disposition

A total of 220 subjects were randomized into the study and all received placebo or RSV F vaccine concurrent with TIV on day 0 (Fig. 1); 90 to 95% of subjects across treatment groups received the rescue TIV (Groups A to D) or second dose of placebo saline (Group E) on day 28. The discontinuation rate was 3 to 5% across the treatment groups, with no discontinuations known to be due to an AE. From 88 to 95% of subjects in each group completed the study without major deviations (PP population).

### Demographic and other baseline characteristics

Baseline characteristics for the study population are presented in Table 1. There were slightly more women than men enrolled across most treatment groups, and a majority of subjects were White or Caucasian (93 to 100%), and not of Hispanic or Latino origin (93 to 100%). Subject mean ages ranged from 67.7 to 69.1 years, with a range of 60 to 87 years, in accordance with the protocol. The distribution of subjects in the 60 to < 75 and the ≥ 75 age strata was identical across the five treatment groups.

**Table 1:** Subject characteristics

Group:	E	A	B	C	D
Characteristic	Placebo *N* = 60	60 μg + Al *N* = 40	60 μg *N* = 40	90 μg + Al *N* = 40	90 μg *N* = 40
Gender, n (%)					
Male	22 (37)	18 (45)	16 (40)	21 (53)	17 (43)
Female	38 (63)	22 (55)	24 (60)	19 (48)	23 (58)
Race, n (%)					
White or Caucasian	58 (97)	40 (100)	39 (98)	37 (93)	40 (100)
Black or African American	1 (2)	0	0	3 (8)	0
Asian	0	0	0	0	0
American Indian/Alaska Native	0	0	0	0	0
Native Hawaiian/Other Pacific Islander	0	0	0	0	0
Other	1 (2)	0	1 (3)	0	0
Ethnicity, n (%)					
Hispanic or Latino	0	0	1 (3)	2 (5)	3 (8)
Not Hispanic or Latino	60 (100)	40 (100)	39 (98)	38 (95)	37 (93)
Age Group (years), n (%)					
60 to < 75	51 (85)	34 (85)	34 (85)	34 (85)	34 (85)
≥ 75	9 (15)	6 (15)	6 (15)	6 (15)	6 (15)
Age (years)					
n	60	40	40	40	40
Mean (SD)	69.1 (5.5)	69.1 (5.8)	67.7 (5.6)	68.0 (5.8)	68.7 (5.7)
Median	68.0	68.0	67.0	68.0	68.0
Min, Max	60, 87	60, 83	60, 85	60, 85	61, 81

Percentages are based on the number of subjects in the Safety Population with non-missing data within treatment group

Age is calculated as the closest integer result of (Date of Study day 0 – Date of Birth)/365.25

### Safety

All RSV F vaccine formulations were well-tolerated, with no deaths, vaccine-related SAEs, or evidence of systemic toxicity pertaining to renal or hepatic injury, or hematologic changes. The frequency and severity of unsolicited AEs, MAEs, SNMCs, and SAEs failed to demonstrate any association of active vaccine with adverse events in any particular body system or diagnosis (Table 2).

**Table 2:** Overview of solicited and unsolicited adverse events

Group:	E	A	B	C	D
AE Category Severity/Relationship	Placebo *N* = 60	60 μg + Al *N* = 40	60 μg *N* = 40	90 μg + Al *N* = 40	90 μg *N* = 40
Verbatim Term	*n* (% of Subjects with Events)				
Local Solicited AEs^a^	14 (23)	17 (43)	9 (23)	17 (43)	15 (38)
Severe	0	0	0	0	0
Pain	14 (23)	17 (43)	8 (20)	17 (43)	10 (25)
Swelling	2 (3)	4 (10)	1 (3)	3 (8)	6 (15)
Bruising	1 (2)	2 (5)	1 (3)	1 (3)	4 (10)
Redness	0	0	1 (3)	2 (5)	3 (8)
Solicited Systemic AEs^a^	22 (37)	12 (30)	6 (15)	16 (40)	10 (25)
Severe	1 (2)	1 (3)	0	0	1 (3)
Muscle Pain	6 (10)	6 (15)	4 (10)	7 (18)	5 (13)
Joint Pain	4 (7)	4 (10)	2 (5)	1 (3)	4 (10)
Headache	10 (17)	2 (5)	5 (13)	9 (23)	3 (8)
Fatigue	12 (20)	4 (10)	2 (5)	6 (15)	8 (20)
All Unsolicited AEs^b^	36 (60)	19 (48)	23 (58)	19 (48)	22 (55)
Related	6 (10)	1 (3)	0	1 (3)	4 (10)
Severe	4 (7)	2 (5)	3 (8)	2 (5)	0
Severe/Related	0	0	0	0	0
Severe or Related	10 (17)	3 (8)	3 (8)	3 (8)	4 (10)
Upper Respiratory Tract Infection	6 (10)	2 (5)	3 (8)	1 (3)	2 (5)
Back Pain	1 (2)	1 (3)	1 (3)	4 (10)	2 (5)
Myalgia	0	0	1 (3)	2 (5)	3 (8)
Oropharyngeal Pain	1 (2)	3 (8)	1 (3)	1 (3)	0
Hypertension	1 (2)	2 (5)	0	2 (5)	1 (3)
Systolic Hypertension	1 (2)	1 (3)	2 (5)	0	2 (5)
SAEs^b^	3 (5)	2 (5)	3 (8)	3 (8)	1 (3)
MAEs^b^	21 (35)	11 (28)	14 (35)	8 (20)	14 (35)
SNMCs^b^	4 (7)	2 (5)	5 (13)	2 (5)	4 (10)

- Percentages are based on the number of subjects in the Safety Population with the event shown. Subjects with multiple events within a category were counted only once, using the event with the greatest severity (mild, moderate, severe) and/or relationship (possible, probable, definite). An AE was considered treatment-emergent if it began on or after the day 0 vaccination

^a^Only includes solicited AEs with an onset within 7 days of the day 0 vaccination. All solicited events were deemed to be related to the test article administered

^b^Includes unsolicited AEs with an onset from days 0 to 56; and significant new medical conditions (SNMCs), medically-attended adverse events (MAEs), and serious adverse events (SAEs) with an onset from days 0 to 364. The unsolicited AEs shown are those that occurred in > 2% of subjects (i.e., 4 or more) in an RSV F vaccine group

The only unsolicited AEs occurring in > 2% of RSV F vaccine recipients were upper respiratory tract infection, myalgia, back pain, oropharyngeal pain, hypertension, and systolic hypertension, which, except for myalgia, occurred with generally similar frequencies in the placebo group. The rescue immunization with TIV was well-tolerated and was not associated with increased frequency or severity of AEs in recipients relative to the placebo group administered TIV at the first vaccination (data not shown).

As expected, solicited local injection site AE reports were modestly more frequent in adjuvanted vaccine recipients at both antigen dose levels (60 and 90 μg) and in 90 μg unadjuvanted vaccine recipients when contrasted with placebo recipients. Pain was the most commonly reported injection site complaint, occurring 2-times more frequently in adjuvanted vaccine recipients compared to unadjuvanted vaccine and placebo recipients. There were no differences in solicited systemic AE complaints, as the most frequently reported events (muscle pain, headache, fatigue, and joint pain) occurred in active vaccine and placebo recipients at closely similar rates. Fever was also infrequently reported, occurring in only one 90 μg unadjuvanted vaccine recipient after the day 0 vaccination, peaking at < 39 °C, and resolving spontaneously.

The majority of AEs reported overall were mild to moderate in severity, with amelioration or resolution of nearly all severe events by study end (through Day 364). Approximately 10% or fewer subjects in any group had an unsolicited event that was deemed possibly related to the study treatment by the investigators; and there were no events that were both severe and related.

### Immunogenicity

**Fig. 2:**
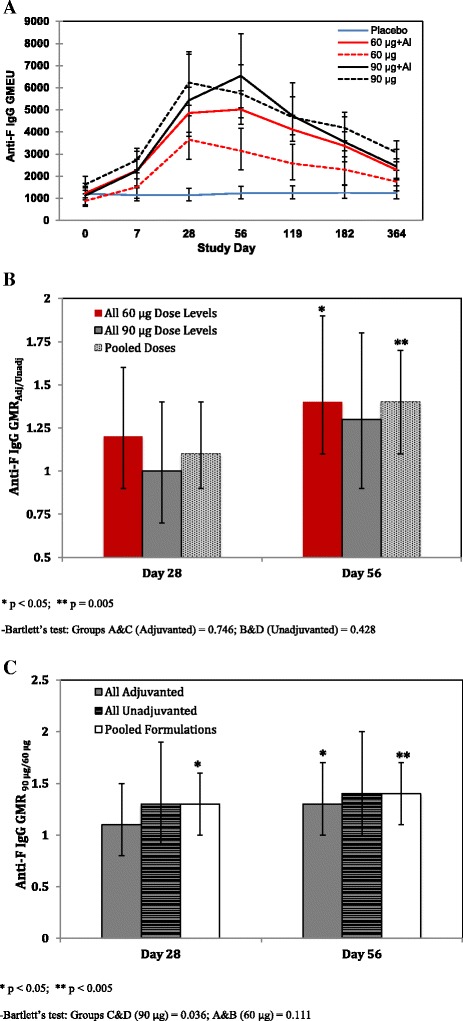
Anti-F IgG response summary and comparative analysis to assess for an adjuvant and antigen dose-effect. **a** Anti-F IgG response kinetics in active vaccine and placebo recipients co-administered TIV. Data are represented by the GMEU and 95% CIs, calculated as the antilog of the mean and 95% confidence limits of log_10_-transformed anti-F IgG EU values. EU values below the assay lower limit of quantitation (LLOQ) of 400 were set to half LLOQ for the purposes of calculation. **b** Demonstration of an adjuvant effect based on the GMR_Adjuvant/Unadjuvanted_ analysis of anti-F IgG EUs on days 28 and 56 in individual groups administered 60 (*red bars*) or 90 μg (*black bars*) RSV F doses or pooled groups (60 and 90 μg, hatched gray bars), with or without adjuvant. Results indicated as significant by single (*p* = 0.016) or double (*p* = 0.005) asterisks allow rejection of the null hypothesis of GMR_Adjuvant/Unadjuvanted_ = 1. **c** Antigen dose-effect analysis based on the GMR_90 μg/60 μg_ of anti-F IgG GMEUs on days 28 and 56 of 90 or 60 μg RSV F recipients of adjuvanted (*black bars*) or unadjuvanted (*black striped bars*), or adjuvanted and unadjuvanted (*pooled, white bars*) vaccines. Results indicated as significant by single (*p* = 0.05), double (*p* = 0.022), or triple (*p* = 0.002) asterisks allow rejection of the null hypothesis of GMR_90 μg/60 μg_ = 1

Anti-F IgG was universally present in all subjects on day 0, but at varying levels indicating that the immunological memory elicited by natural RSV infection is heterogeneous. Post-vaccinal results with all 60 and 90 μg RSV F vaccine formulations, with and without the aluminum phosphate adjuvant, demonstrated a rapid rise in anti-F IgG levels by day 7, consistent with a “booster” response in a primed population, that reached peak levels of 4- to 5-fold higher than baseline at day 28 (unadjuvanted vaccine recipients) or day 56 (adjuvanted vaccine recipients) (Fig. 2a). Anti-F IgG levels remained elevated through six months post-vaccination in active vaccine recipients, but generally declined to less than half of the peak response. Both peak anti-F IgG responses and their durability over time appeared superior in the two adjuvanted treatment groups and the 90 μg RSV F unadjuvanted group relative to the 60 μg RSV F unadjuvanted group. Anti-F IgG levels in the placebo group remained unchanged from baseline.

Two important study objectives were to determine whether an adjuvant effect could be demonstrated and whether the 90 μg antigen dose could show an immunogenicity advantage over the 60 μg antigen dose. Aluminum phosphate adsorption of the RSV F antigen resulted in a statistically significant enhancement of the anti-F IgG response in pooled antigen dose groups (1.4-fold increase on day 56, *p* = 0.005) relative to the pooled unadjuvanted vaccine groups at day 56, although not day 28. This difference was primarily attributable to enhancement of the anti-F IgG response at the 60 μg antigen dose level, which individually showed a significant effect (Fig. 2b). As shown in Fig. 2c, the 90 μg antigen dose produced stronger responses, with or without aluminum phosphate, relative to the 60 μg dose, eliciting a near-significant 1.4-fold increase in response to unadjvuanted formulations (p = 0.06) and a significant 1.3-fold increase in response to adjvuanted formulations (p = 0.022,) on day 56. Pooled analyses showed significant 1.3-1.4 fold RSV-F dose effects on anti-F IgG responses on both days 28 and 56.

**Fig. 3:**
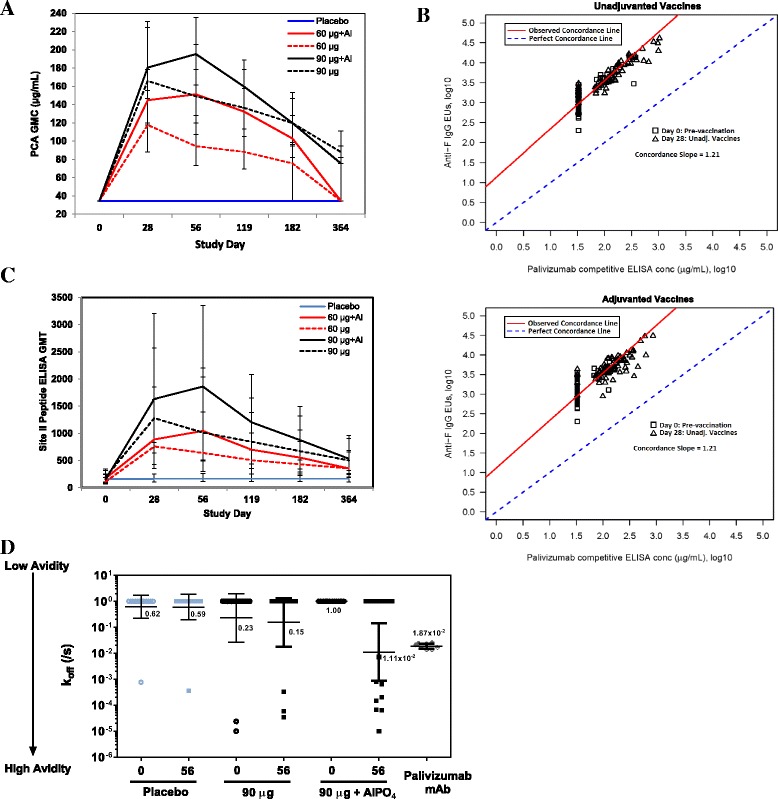
Antibody responses to the antigenic Site II epitope of the RSV F protein. Antibody response kinetics based on PCA (μg/mL with 95% CIs, **a**) and Site II Peptide (GMT with 95% CIs, **c**) ELISAs in active vaccine and placebo recipients co-administered TIV. Scatter plots (**b**) of anti-F IgG EU versus PCA concentration on pre-immune sera (baseline, day 0) from all groups and on day 28 sera from placebo recipients (squares, both figures) and pooled unadjuvanted (triangles, top figure) or adjuvanted (triangles, bottom figure) RSV F vaccine recipients. The hatched blue line denotes the perfect concordance, while the solid red line denotes the observed concordance. Surface plasmon resonance with antigenic Site II peptide (**d**) using days 0 and 56 sera obtained from unadjuvanted or aluminum phosphate adjuvanted, 90 μg RSV F vaccine recipients, or placebo recipients (*N* = 15 subjects per group); or using a palivizumab control (*n* = 8 replicates). All data points in the entire group were used to calculate the geometric mean k_off_

Results of the PCA analysis, which detects antibodies with specificity to an epitope shown to be clinically relevant in the prevention of RSV disease, largely followed the kinetic pattern of anti-F IgG (Fig. 3a). The post hoc concordance analysis [11] further demonstrates the close relationship of both antibody responses after active vaccination (Fig. 3b), but not in placebo recipients. Notably, PCA levels were not measurable above the lower limit of quantitation (LLOQ, 33 μg/ml) at day 0 in 88% of subjects, but rose rapidly from baseline in all active vaccine groups, indicating subjects were immunologically primed to this epitope. PCA levels declined one year post-dosing, but remained above the LLOQ in ~ 65% of 90 μg adjuvanted and unadjuvanted vaccine recipients. The antigenic Site II peptide ELISA, which measures binding of vaccine-induced IgG antibodies to the linear peptide (amino acids 254–278) of the F protein that is recognized by palivizumab, followed a very similar pattern of rise and decay when compared to the PCA antibody kinetics for all formulations (Fig. 3c). Surface plasmon resonance (SPR) analysis, performed post hoc to access the binding avidity of vaccine-induced antibodies to the immobilized antigenic Site II peptide (Fig. 3d), found that few subjects had high avidity antibodies at day 0. Following immunization, there was a net gain by day 56 of 1 subject with high avidity antibodies in the unadjuvanted vaccine group, compared to a net gain of 8 subjects with high avidity in the adjuvanted vaccine group.

**Fig. 4:**
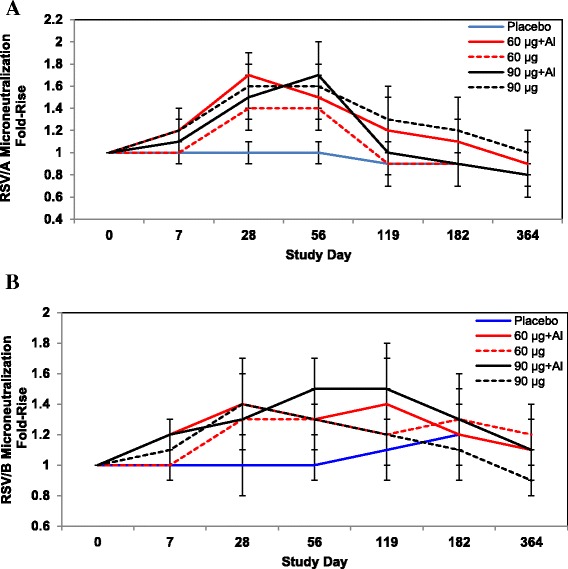
Summary of RSV/A and RSV/B Microneutralization Responses. RSV/A (**a**) and RSV/B (**b**) microneutralization antibody response kinetics in active vaccine and placebo recipients co-administered TIV based on the fold-rise in post-vaccination GMTs relative to baseline (GMR_Post/Pre_). Data are represented by the GMT and associated 95% CIs. The pooled day 0 GMT was log_2_ 8.2 or GMT 294 (258–334 95% CI) for RSV/A MN and log_2_ 8.1 or GMT 284 (239–336, 95% CI) for RSV/B MN

The pooled day 0 RSV/A and RSV B MN GMTs were log_2_ 8.2 and log_2_ 8.1 respectively, but varied considerably by group (data not shown). Given the relatively high day 0 antibody titers, the magnitude of the post-vaccinal MN fold-rise was less than those observed in the anti-F IgG, PCA, and antigenic site II ELISAs, and lower than the increase previously achieved in young healthy adults and women of childbearing age [9, 10]. Peak fold-increases in MN titer attained on day 28 (unadjuvanted groups) or day 56 (adjuvanted groups) showed little differentiation across formulations for RSV/A (1.4- to 1.7-fold increases) or RSV/B (1.3- to 1.5-fold increases) (Fig. 4), and did not achieve statistical significance relative to the placebo group. Three placebo subjects did attain a ≥ 4-fold increase in MN titer when sera were sampled after day 56 (data not shown), the timing of which coincided with the RSV season and is suggestive of RSV exposure.

**Fig. 5:**
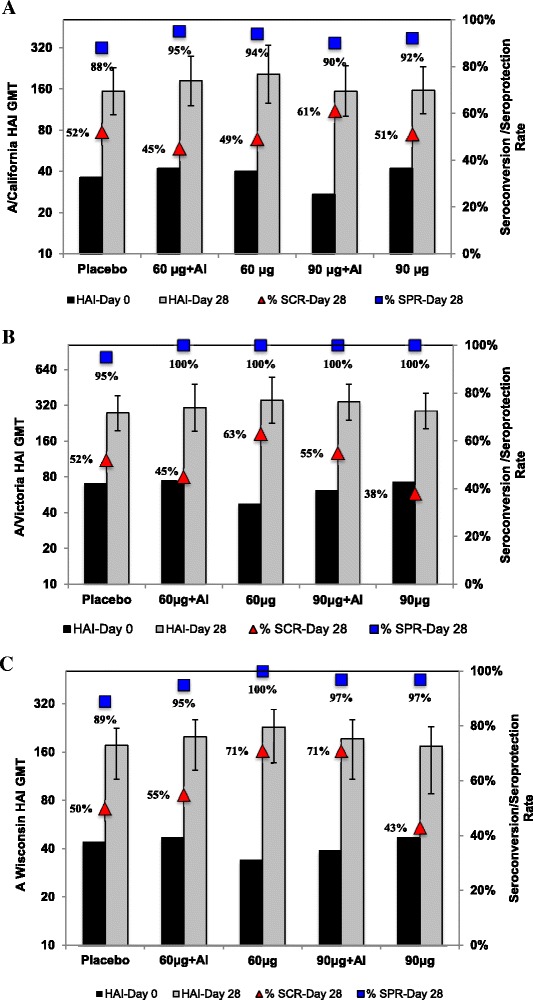
Influenza HAI titers in TIV co-administered RSV F vaccine and placebo recipients. HAI GMTs (left axis) with associated 95% CIs to the A/California (**a**), A/Victoria (**b**), and A/Wisconsin (**c**) vaccine strains in each treatment group at day 0 (*black bars*) or day 28 (*gray bars*). Point estimates of day 28 HAI seroconversion (*red triangles*) and seroprotection (*blue squares*) rates by treatment group are also shown (*right axis*)

Given that RSV and influenza share a common seasonality and that co-immunization with vaccines developed against both infectious agents is cogent from a logistics and compliance perspective, it was important to seek evidence of interference with resultant influenza HAI responses with this strategy. Because this initial Phase 1 study was not powered to test for non-inferiority, the HAI analysis provided only a descriptive summary of vaccine responses among the RSV F/TIV groups versus the placebo/TIV group. As shown in Fig. 5, there was no apparent negative impact of RSV F co-administration on influenza HAI titers achieved, as post-immunization GMTs for all antigens were comparable to, or higher than, those of the placebo group. Rates of seroprotection, defined as an HAI titer ≥ 40, were ≥ 90% in all co-administered groups for all influenza vaccine antigens. Rates of seroconversion, defined as baseline HAI titer < 10 and a post-vaccination HAI titer ≥ 40, or a baseline titer ≥ 10 and a 4-fold increase in post-vaccination titer were more variable in the co-administered groups (45 to 61% for A/California; 38 to 63% for A/Victoria; and 43 to 71% for B/Wisconsin on day 28), but were not systematically less than rates observed for placebo recipients (52%, 52%, and 50% for each respective virus on day 28).

## Discussion

In this study, we evaluated a novel RSV F vaccine for the first time in an older adult population. The RSV F vaccine was well-tolerated and highly immunogenic across all doses and formulations tested, eliciting rapid, booster-like post-vaccinal increases in vaccine-specific antibodies, including strikingly concordant increases in anti-F IgG and PCA (Fig. 3b). In comparison, the high baseline levels of neutralizing antibody only resulted in a modest boost in titers post-vaccination. We have reported this phenomenon previously in young adults; and have shown, given the relatively high background levels of RSV MN antibodies in normal adult sera, that adding even fairly large concentrations of the known neutralizing monoclonal palivizumab (80–120 μg/mL) results in only a modest fold-increase in MN titers [9]. Nevertheless, all measured immune responses were durable over a period consistent with potential protection for a temperate winter RSV transmission season, but manifested a substantial decline over one year of follow-up.

As in younger adults [9, 10], PCA levels in pre-immune sera were notably low (generally below the assay LLOQ of 33 μg/mL) in this population, despite evidence of high baseline MN antibody titers indicating a history of previous RSV/A and RSV/B infections. The presence of day 0 Site II peptide antibodies provide further evidence that this antigen is presented to the immune system during infection. It seems likely that PCAs are present at low levels in elderly persons, but below the LLOQ of the currently available assay. Alternatively however, it is also possible that immunization with the RSV F vaccine fundamentally changes the spectrum and proportion of antibodies in serum with palivizumab-like specificities. In other words, the vaccine may induce a class of binding specificities which do not occur at significant levels after natural RSV infection, despite multiple lifetime exposures. Along these same lines, aluminum phosphate adjuvantation appears to provide both a quantitative and qualitative enhancement in the immune response produced by older adult vaccine recipients, as evidenced by both the increase in Site II-specific antibodies and the fact that a substantial proportion of these antibodies (greater than with unadjuvanted vaccine alone) bind to the Site II epitope of the F protein with high avidity (Fig. 3d). It is possible to speculate that induction of high avidity Site II antibodies might contribute importantly to protection, and that immunization with an aluminum phosphate-adjuvanted RSV F vaccine as a two-dose regimen might further improve the affinity maturation of anti-F specific antibodies [12].

PCA concentrations and MN titers rise in parallel after RSV F immunization and are associated with protection against RSV challenge in animals models (e.g., cotton rats) that lack background levels of RSV-specific neutralizing antibodies [7, 8]. In the absence of data from prospective, well-designed, controlled efficacy trials, the correlation of vaccine-induced neutralizing antibodies and other frequently measured immune measures to RSV with clinical protection remains to be defined [13]. Although sero-epidemiology studies in both children and adults have shown an inverse relationship between RSV-specific serum neutralizing antibody levels and the risk of RSV-associated hospitalization [14, 15], these findings do not fully explain the ongoing risk of recurrent RSV infection as MN levels are often high in susceptible populations [16]. In a surveillance study conducted during a single RSV season, Falsey et al. determined there was an inverse relationship between RSV-neutralizing antibody titer derived from past infections and disease risk [17], although there was considerable overlap between MN titers of infected and uninfected individuals. In older adult humans, anti-RSV immune responses following decades of recurrent natural RSV exposure include a complex array of anti-F, anti-G, and other antibody specificities which may or may not be protective despite activity in an in vitro neutralization assay. This quantitatively substantial milieu of baseline circulating RSV neutralizing antibodies may not, at a given level of neutralization antibody titer, consistently and qualitatively indicate clinical protection. Data from substantial controlled trials will be required to understand the contribution of vaccine-induced immune responses.

Finally, given the winter seasonal nature of RSV transmission in temperate regions and the observed decline of RSV F vaccine-specific antibody responses over the course of a year of follow-up, the RSV F vaccine, if shown to be safe and efficacious, would likely be administered prior to the onset of winter along with a seasonal influenza vaccine. In preliminary support of potential future strategies to co-administer RSV F and seasonal influenza vaccines, we demonstrated the absence of an adverse impact of RSV F and seasonal influenza vaccine co-administration on HAI responses.

## Conclusions

Preliminary clinical development of this novel RSV F vaccine in older adults indicates an acceptable safety profile and the induction of neutralizing anti-RSV immune responses that include responses specific for antigenic Site II, which is known to be the target of protective antibodies in children. The substantial burden of severe RSV disease in older adults and the predictable nature of the annual RSV epidemic warrant further development of this promising vaccine to address an urgent unmet public health gap.

## Abbreviations

AE: Adverse event
ANCOVA: Analysis of covariance
EU: ELISA units
F: Fusion protein
GM: Geometric mean
GMC: Geometric mean concentration
GMEU: Geometric mean ELISA unit
GMR: Geometric mean ratio
GMT: Geometric mean titer
HAI: Hemagglutination inhibiting antibody
IM: Intramuscular
LLOQ: Lower limit of quantification
MAE: Medically-attended events
PCA: Palivizumab-competitive antibodies
PP: Per protocol
PT: Preferred term
RSV: Respiratory syncytial virus
SAE: Serious adverse event
SCR: Seroconversion rates
SNMC: Significant new medical conditions
SOC: System organ class
SPR: Seroprotection rates
TIV: Trivalent influenza vaccine
